# Otitis Media in Infancy and Early Childhood

**Published:** 1907-11-16

**Authors:** 


### OTOLOGY.

### OTITIS MEDIA IN INFANCY AND EARLY CHILDHOOD.

### THE IMPORTANCE OF INSPECTION OF THE TYMPANIC MEMBRANE.

ALTHOUGH suppurative otitis media may occasionally subside spontaneously, otorrhœa in infants must be looked upon as a grave affection. In the first place the risk of meningitis complicating acute middle ear inflammation is greater in infants and children than it is in adults. Secondly, otorrhœa is extremely apt to recur or persist, and to become associated with dangerous complications of chronic suppurative otitis media—namely, extra-dural abscess, lateral sinus thrombosis, meningitis, and brain abscess. Thirdly, apart from these intracranial complications, the general nutrition of the child may be profoundly affected; gastro-intestinal disorders may outrun the local disease, which may terminate fatally from exhaustion and malnutrition associated therewith. Fourthly, should the child escape progressive disease of the middle ear and these complications, the function of hearing and the mental development of the child may be impaired. Lastly, there is no doubt that the causes of disturbances of hearing which we meet with in after-life are frequently to be sought for in the overlooked or disregarded affections of the ear occurring in infancy and early childhood.

In the acute stage the stress of the inflammation falls upon the mucosa. The fine connective tissue network of the mucosa is infiltrated with leucocytes and proliferated connective tissue cells, and but little change is met with in the bone. After a time absorption of the bone trabeculæ can be demonstrated histologically, so that the marrow cells and sub-epithelial layers of the mucosa are thrown into direct continuity. Of the organisms met with, streptococci are the commonest.

#### SYMPTOMS.

In infants otitis media begins sometimes with little or no evidence of pain, or other obvious local symptom, particularly in children with adenoids; the symptoms may be masked in the specific fevers. Restlessness and crying are always suggestive of discomfort or pain. In an apparently healthy infant gastro-intestinal disturbances are no doubt most frequently to blame, but the ears should in dubious cases always be examined and the tympanic membrane inspected. It is not commendable to wait for the appearance of otorrhœa before diagnosing otitis media. Restlessness, constant crying, rolling of the head, sometimes, but not always, the placing of the hands to the ears or head: these are the signs of ear-ache in infants. We may further observe the effect of warmth applied first to one and then to the opposite ear, in soothing the child, or he may show an obvious preference for a dependent position of the head to the side most affected.

Sometimes pyrexia unattended by pain is the outstanding feature of the disease. When fever is accompanied by vomiting, convulsions, and loss of consciousness, we are often unable at first to state the nature of the cause, unless evidence of otitis media is discovered by inspecting the tympanic membrane. So-called meningeal irritation is not rare in young subjects suffering from acute otitis, and the symptom-complex may point to meningitis, though no meningitis exist, for the meningeal symptoms may completely subside with the appearance of pus in the meatus.

The discharge is often profuse. It may be slightly blood-stained, and is sometimes so irritating to the skin of the meatus and concha that pustules appear and break, leaving an ulcerating surface, covered with scabs. This pustular condition is aggravated by wool placed in the ear to "soak up the discharge."

The results of neglect are more frequently seen among patients in the humbler walks of life. The idea is still all too prevalent that the purulent discharge is one of the incidents commonly associated with teething, that it is inevitable, that it will cease only when the eruption of the milk teeth is completed. Very frequently no treatment is attempted beyond a perfunctory wiping away of all visible discharge, probably with a sponge.

### Otoscopic Appearances.

In the acute stage hyperæmia is the first change visible. Peripheral vessels around the margin of the membrane and those over the handle of the malleus dilate, so that the whole membrane is injected scarlet, the surface may retain lustre, though more often it becomes dull from the collection of an exudate on its surface. Soon the normal landmarks are obscured, and the upper and posterior part of the membrane bulges into the meatal canal, as the inflammatory products collect in the tympanum. This bulge is very typical when the condition of the membrane is observed from the outset, and when the inflammation ends in suppuration. Before perforation takes place a pale conical eminence may be seen on the membrane. The perforation may be so minute that only the thinner, more watery contents escape, while the thick mucus is pent up within the tympanic cavity. It is of the highest importance to bear in mind that in infants and young children such a condition of the membrane with a minute perforation and a distended tympanum may persist for days or weeks, or even months, accompanied by otorrhœa. Unless the drum is properly inspected, it is quite impossible to ascertain whether the perforation is small or large, whether the membrane bulges or not, and whether the condition is favourable for whatever line of treatment is being adopted.

In cases in which granulations form, or in which the perforation is large and unaccompanied by distension of the drum, myringotomy would not be indicated.

### Treatment.

In cases of bulging membrane without other local physical signs, the line of treatment is clear. No matter whether there be a perforation or not, if the membrane bulges it denotes over-distension of the tympanic cavity. The membrane should be freely incised, and if this is done the probability is that the otorrhœa will cease and the incision heal completely in ten days or a fortnight. This desirable result has been observed not only when the otorrhœa has been recent in origin, but also after it has persisted for two and for three months.

The incision in the membrane should be made with all the principles of surgical cleanliness. An anæsthetic should be administered. Formerly I invariably preferred general anæsthesia, but I find Hechinger's solution,* when fresh, will produce complete anæsthesia for this operation in about seven minutes. The fluid should be applied as drops to fill the meatus, which has been previously cleansed. We should bear in mind the object of the incision, namely, to give a free exit for the tympanic contents. The after treatment is directed to the prevention of secondary infection of the tympanum and the removal of the inflammatory exudate by syringing with non-irritating antiseptic fluid, miscible with mucus. The following alkaline lotion fulfils these requirements:

| | |
|---|---|
| Sodium bicarbonate ... ... ... | grs. x. |
| Sodium biborate ... ... ... | grs. x. |
| Glycerine ... ... ... ... | ♏xxx. |
| Normal saline solution ... ... | to 1 oz. |

The fluid must be used warm, after adding an equal quantity of hot water. The syringing should be repeated from three to six times daily, according to the profuseness of the discharge. No wool should be placed in the concha or meatus. It is better to apply an antiseptic powder composed of equal parts of borax, zinc oxide, and starch. In conclusion if the tympanic membrane be inspected in every case of otorrhœa in infants the condition of bulging membrane with minute perforation is very commonly met with; of the cases treated by syringing alone without incision only a small proportion are cured, whereas the results of myringotomy, even when the condition is chronic, are exceedingly favourable, and complete recovery may be anticipated.

| | |
|---|---|
| * Ac. Carbol. Liq. ... ... ... | 1 part |
| Cocain. Hydrochlor. ... ... ... | 2 parts |
| Menthol ... ... ... ... ... | 2 parts |
| Spiritus Vini Rect. ... ... ... | 20 parts |

